# Degenerative Cervical Myelopathy: An Overview

**DOI:** 10.7759/cureus.50387

**Published:** 2023-12-12

**Authors:** Laura M Saunders, Hushil S Sandhu, Lorcán McBride, Vindhya S Maniarasu, Samantha Taylor, Rakesh Dhokia

**Affiliations:** 1 School of Medicine, Dentistry and Biomedical Sciences, Queen’s University Belfast, Belfast, GBR; 2 Department of Trauma and Orthopaedics, Royal Victoria Hospital, Belfast, GBR; 3 Department of Medicine, Royal Infirmary Hospital, Edinburgh, GBR

**Keywords:** literature review, spinal surgery complication, posterior cervical decompression and fusion, anterior cervical discectomy fusion, musculoskeletal imaging, modified japanese orthopaedic association score, surgical management of spine, anatomy, degenerative cervical myelopathy

## Abstract

Degenerative cervical myelopathy (DCM) is a spinal condition of growing importance due to its increasing prevalence within the ageing population. DCM involves the degeneration of the cervical spine due to various processes such as disc ageing, osteophyte formation, ligament hypertrophy or ossification, as well as coexisting congenital anomalies. This article provides an overview of the literature on DCM and considers areas of focus for future research.

A patient with DCM can present with a variety of symptoms ranging from mild hand paraesthesia and loss of dexterity to a more severe presentation of gait disturbance and loss of bowel/bladder control. Hoffman’s sign and the inverted brachioradialis reflex are also important signs of this disease.

The gold standard imaging modality is MRI which can identify signs of degeneration of the cervical spine. Other modalities include dynamic MRI, myelography, and diffusion tensor imaging. One important scoring system to aid with the diagnosis and categorisation of the severity of DCM is the modified Japanese Orthopaedic Association score. This considers motor, sensory, and bowel/bladder dysfunction, and categorises patients into mild, moderate, or severe DCM.

DCM is primarily treated with surgery as this can halt disease progression and may even allow for neurological recovery. The surgical approach will depend on the location of degeneration, the number of cervical levels involved and the pathophysiological process. Surgical approach options include anterior cervical discectomy and fusion, corpectomy, or posterior approach (laminectomy ± fusion). Conservative management is also considered for some patients with mild or non-progressive DCM or for patients where surgery is not an option. Conservative treatment may include physical therapy, traction, or neck immobilisation.

Future recommendations include research into the prevalence rate of DCM and if there is a difference between populations. Further research on the benefit of conservative management for patients with mild or non-progressive DCM would be recommended.

## Introduction and background

This overview will focus on the anatomy, clinical presentation, diagnosis, and management of the cervical spine affected by degenerative cervical myelopathy (DCM). The primary aim of this report is to summarise, build upon, and critically appraise the literature on DCM to date. While the current literature has contributed to this subject area, there are still gaps in the knowledge about DCM. For example, there is a need for more definitive diagnostic tools and criteria to identify patients with DCM. The natural history and progression of DCM are poorly understood and therefore a need for more longitudinal studies to track this. There is also little evidence of DCM prevalence globally and possible regional differences. There are few randomised control trials to assess and compare the efficacy of various treatments such as surgical interventions and conservative treatment.

The anatomy of the cervical spine

Osteology

**Figure 1:**
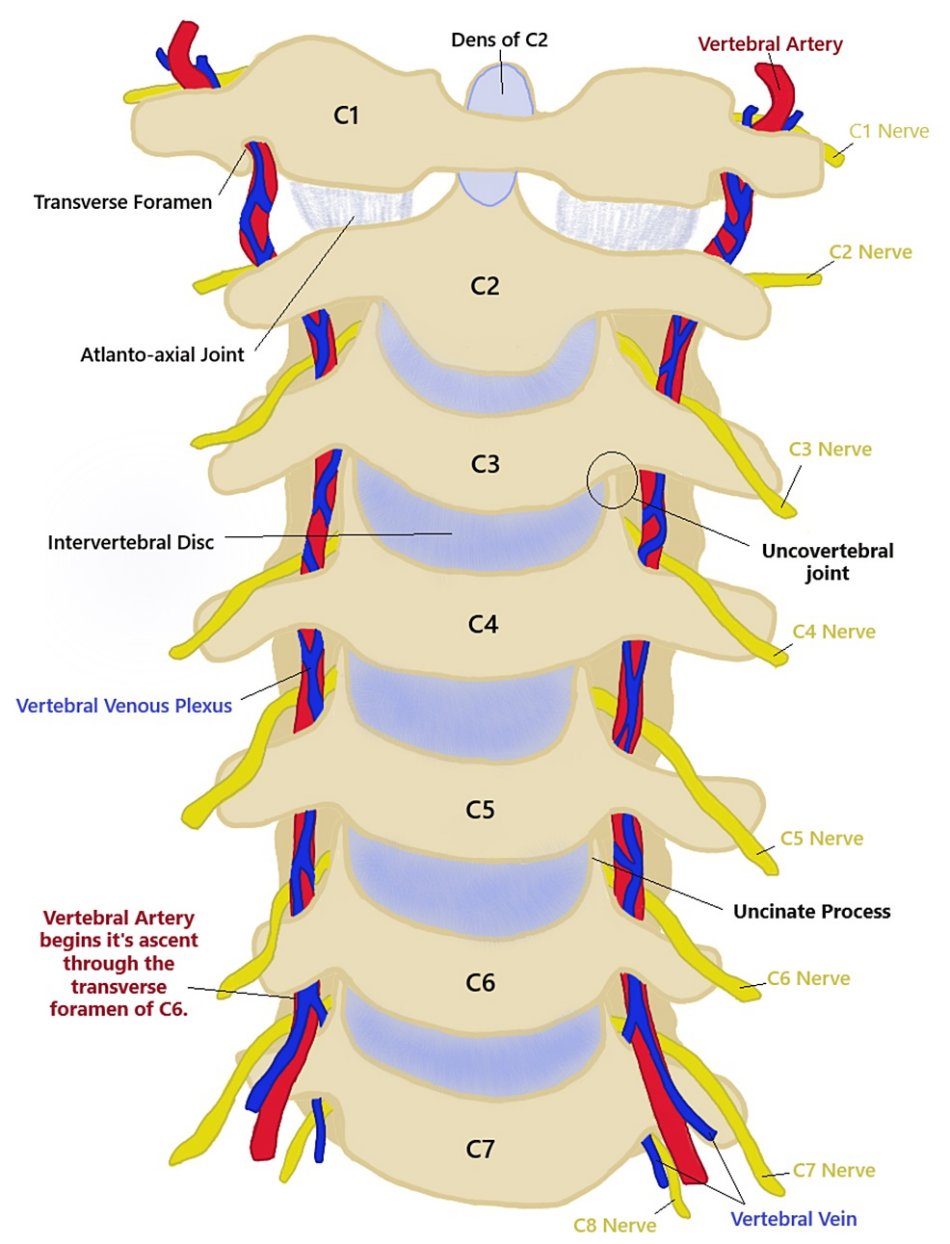
A drawing of the cervical spine anatomy including blood supply and nerve location. This is the original work of the authors.

The cervical spine is composed of seven vertebrae with eight associated spinal nerves. C1 and C2 are atypical vertebrae and have no intervertebral (IV) disc between them (Figure 1). The transverse process of cervical vertebrae contains the transverse foramen which transmits the vertebral artery, vertebral vein, sympathetic plexus, and spinal nerves. The IV discs between the vertebrae are composed of tough annulus fibrosis surrounding a fibro gelatinous nucleus pulposus [1]. The IV discs act as a fulcrum and shock absorber during dynamic movement of the cervical spine. The cervical discs are the thinnest out of all IV discs; however, they have the greatest relative thickness to the size of the corresponding vertebral body [2]. Furthermore, the cervical vertebrae are the smallest in size compared to all other vertebrae. The size of the IV discs and vertebral bodies increases in a cranial-caudal direction. It is important to determine the size of the IV disc and vertebral bodies to accurately plan for surgery [3]. Uncovertebral joints are found between the unci of the bodies of C3-C7 and the vertebral bodies superior to them. These joints are important as they are often the site of bone spur or osteophyte formation in the later years of life [4].

Blood Vessels

A notable and unique feature of the cervical spine is the presence of the oval transverse foramina which provide a pathway for the vertebral arteries and veins. The transverse foramen of C7 transmits only the vertebral vein and the vertebral arteries start their ascent through the transverse foramen of C6 [5] (Figure 1). The cervical spine is supplied by a single anterior spinal artery and two posterior spinal arteries. These arteries originate from the vertebral arteries and the posterior inferior cerebellar arteries, respectively. There are also segmental spinal arteries at each spinal level and occasional medullary spinal arteries [6].

Nerves

Cervical spinal nerves are unique as they emerge through the IV foramina superior (except C8) to the corresponding vertebral level after which they are named. The other spinal nerves emerge inferior to their corresponding vertebra level [4,5]. As seen in Figure 1, the C3 spinal nerve emerges between the C2 and C3 vertebrae. The cervical nerves provide innervation to the upper limbs through the brachial plexus (C5-T1). When these nerves are compressed, this can present with radicular symptoms such as muscle weakness, sensory dysfunction, and loss of reflexes [7]. Accessory transverse foramen are generally found posterior to the transverse foramen and can be implicated in the compression of spinal nerves. A study of 250 Turkish dry cervical vertebrae by Ogut et al. [8] found an incidence of the accessory transverse foramen of 8.4% with 76.2% located posteriorly. The most common location for the accessory transverse foramen was C7 (33.3%).

**Figure 2:**
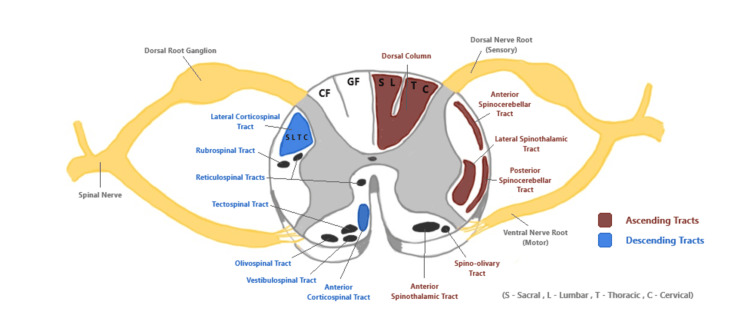
A cross-sectional drawing of the cervical spinal cord. This demonstrates the location of the spinal tracts within the spinal cord that can be affected by degenerative cervical myelopathy. CF = cuneatus fasciculus; GF = gracile fasciculus This is the original work of the authors.

The most important spinal cord tracts affected by spinal cord compression due to DCM are the corticospinal tract, spinothalamic tract, and dorsal column [7] (Figure 2). The corticospinal tract transmits motor signals from the cerebral cortex to distal muscle groups [4]. It is located in both the anterior and lateral white matter. The anterior corticospinal tract is mainly concerned with posture control [4].

The lateral corticospinal tract controls skilled movements of the limbs and can be compressed in DCM. Sacral and lumbar nerve fibres are located laterally in this tract and are often compressed first [9]. This corresponds to the clinical presentation of DCM where lower extremity motor signs are predominant [2]. The dorsal column is responsible for transmitting sensory signals ipsilaterally for discriminative touch, vibration, and proprioception. It can be subdivided into the gracile fasciculus and cuneate fasciculus which transmit sensory information from below T6 and above T6, respectively [9]. As seen in Figure 2, the cuneate fasciculus is located laterally to the gracile fasciculus and is usually compressed first. This corresponds to the clinical presentation of DCM given that sensory disturbance in the hands usually develops early on with this disease [9]. The spinothalamic tract transmits contralateral pain, temperature, and crude touch sensations. It is generally affected along with the lateral corticospinal tract to produce a typical presentation for DCM. Other tracts that can be compressed include the anterior and posterior spinocerebellar tracts located in the lateral white matter. Compression of these tracts contributes to gait disturbance and issues with coordination [9].

## Review

Epidemiology

The epidemiology of DCM is generally poorly understood as there is a paucity of incidence and prevalence rates reported within the literature [2,10]. Inconsistent definitions for DCM have been used in the past by healthcare professionals. Therefore, diagnosis can be difficult due to the variable nature of the disease presentation [11]. In more recent years, there has been a consensus on what constitutes a diagnosis of DCM, which will help guide research in the future [12].

Wu et al. [13] estimated the incidence of DCM in the Taiwanese population as 4.04 to 7.88 per 100,000 people. They also found a peak incidence of 28.98 per 100,000 men and 15.3 per 100,000 women who were >70 years old. This is one of the few studies relating to the incidence and prevalence of DCM. A strength of this study was the use of a comprehensive nationwide database covering more than 99% of the population. This allowed for accurate data collection and longitudinal analysis which likely produced a result that is representative of the entire population. A more recent study in a UK population estimated the incidence to be 7.44 per 100,000 people [14]. This study provided insights into the incidence of DCM based on secondary care admission. It can be used to guide future surgical considerations in the UK. A limitation of this study was that the reported incidence was likely underestimated as patients managed conservatively were not considered. Wu et al. [13] and Goacher et al. [14] reported a similar incidence rate based on hospitalisations for DCM-related injuries. A further study in a UK population estimated the prevalence of DCM to be 2.22 per 100,000 people with a peak incidence of 4.16 per 100,000 in patients aged >79 years [15]. Interestingly, this paper suggested that DCM is underdiagnosed and instead attributed to normal ageing by clinicians. They identified a potential health inequality when they compared DCM prevalence based on spinal cord compression rates and hospital-diagnosed prevalence. The difference between these was greater for older patients; however, the reason for this is unknown. A limitation of this finding is that it was based on estimates which could introduce uncertainty to the result.

DCM typically presents in adults in their 50s and 60s. It is more common in males and tends to present with more severe symptoms with degeneration at multiple cervical levels [2,14,16]. The increased incidence of DCM in males could be explained by occupational and environmental exposures such as heavy lifting and repetitive movements [17]. The prevalence of DCM is expected to increase as the patient demographic shifts with the ageing population and with the advancement of diagnostic techniques [18].

Additionally, an electrophysiological study of 129 patients conducted by Tani et al. [19] found that the upper cervical segments C3-C4 and C4-C5 were more commonly involved with increasing age. Overall, 92% of patients over 70 had C3-C4 and C4-C5 involvement compared to 68% of patients under 60 with predominant C5-C6 and C6-C7 involvement. This study contributes a statistically significant finding and a valuable insight into the age-related shift in the location of DCM involvement. This is a key factor that clinicians could consider for diagnosis and surgical planning. However, a few limitations identified in this study included the small sample size, the absence of radiological findings, and no patient demographics which could significantly influence these results.

The pathophysiology of degenerative cervical myelopathy

The cervical spine is a highly mobile area that is susceptible to degenerative changes, particularly in later life. DCM is a multifactorial disease with numerous factors involved in the disease process. It involves the degeneration of multiple components of the cervical spine such as the IV disc, vertebral body, posterior longitudinal ligament (PLL), ligamentum flavum (LF), and facet joints [20].

**Figure 3:**
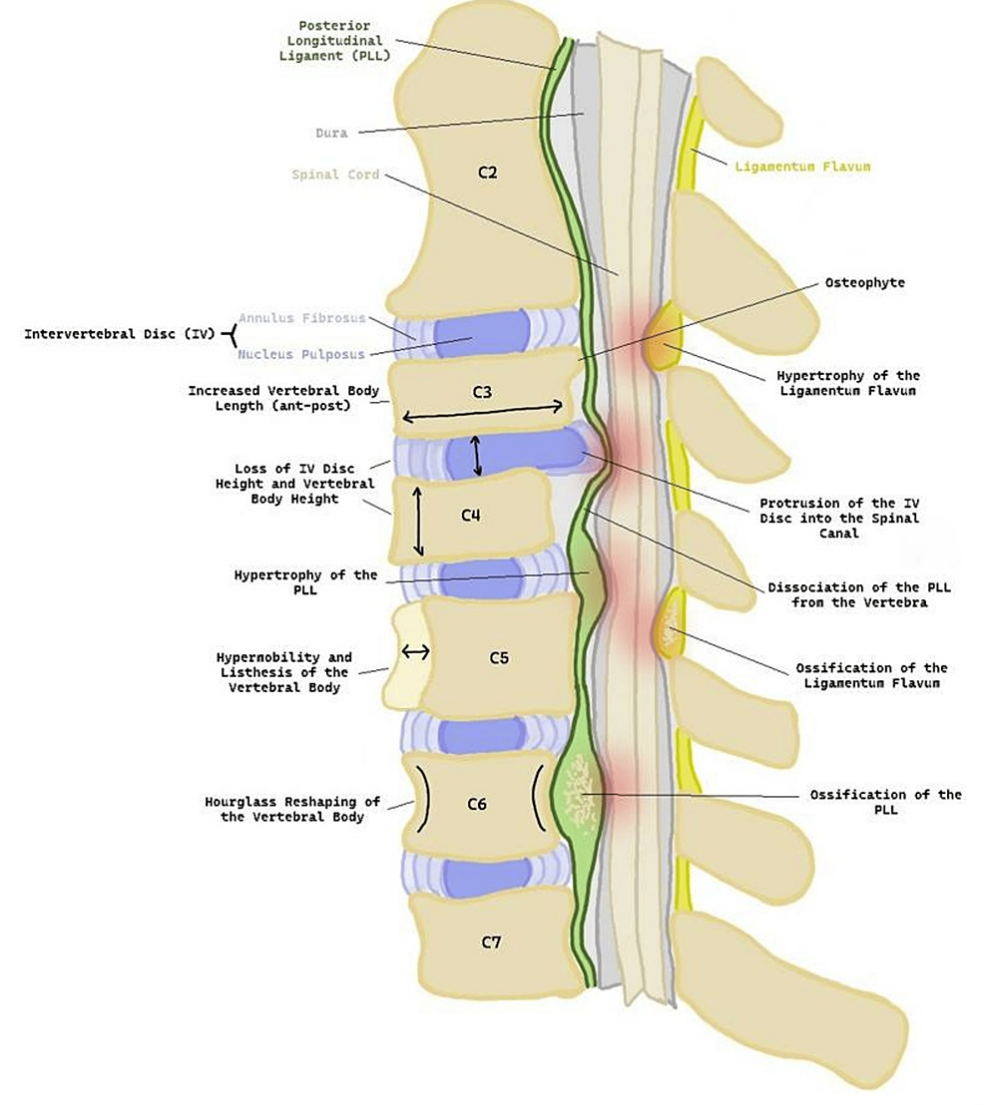
A sagittal drawing of the cervical spine depicting the degenerative changes involved in degenerative cervical myelopathy. Degenerative changes within the cervical spine include changes to the vertebral body such as loss of height, increased anterior-posterior length, and hourglass reshaping. The vertebral body can become hypermobile and lead to spondylolisthesis. Osteophyte formation can occur leading to anterior compression of the spinal cord. The intervertebral (IV) disc can degenerate and lead to loss of IV height and protrusion. The posterior longitudinal ligament (PLL) and ligamentum flavum (LF) can also be affected through hypertrophy and ossification. Points of compression are highlighted in red on the illustration. This is the original work of the authors.

A common age-related degenerative change involved in DCM is cervical spondylosis. This involves age-associated wear and tear that affects the disc, causing disc bulging and loss of joint height, as shown in Figure 3. The disc degenerates and reduces in height due to loss of nutrition, water, and an important proteoglycan called aggrecan [1,21]. The loss of disc height alters the mechanics of the facet joints and can lead to osteophyte formation. At the endplates of the IV disc, there is synovitis, articular cartilage destruction, and eventually joint subluxation [17]. The disc can herniate and cause anterior compression of the spinal cord. The reduction in IV-disc height can also cause buckling of the LF [21]. Overall, this contributes to the narrowing of the spinal canal and neuroforamina which can predispose to cord and nerve root compression leading to myelopathy [1].

The static degenerative changes include ossification of the PLL and hypertrophy of the LF. These can lead to spinal canal narrowing and ultimately cord compression. Other static pathological changes involved in DCM include osteophyte formation, increased vertebral body length, loss of vertebral body height, and desiccation of the disc [21]. The static causes of DCM can be divided into osteoarthritic changes, non-osteoarthritic changes, and congenital changes, as shown in Table 1 [11].

**Table 1:** A summary of the static causes of degenerative cervical myelopathy [11]. IV = intervertebral; PLL = posterior longitudinal ligament; LF = ligamentum flavum

Osteoarthritic changes	Non-osteoarthritic changes	Congenital anomalies
Disease of the IV disc	Ligament hypertrophy (PLL and LF)	Congenital canal stenosis
Osteophytes and bone remodelling	Ossification of the ligaments	Down syndrome
Facet joint involvement		Klippel-Feil syndrome

Ossification of the PLL has now been included as a factor in DCM development as it has been excluded in the past [22]. The sagittal diameter of the spinal canal is typically 17-18 mm between C3 and C7. A diameter of <13 mm is a risk factor for developing cervical myelopathy [23]. Congenital cervical stenosis is indicated when the ratio of the sagittal diameter of the cervical canal to that of the corresponding vertebral body is less than 0.8 [11]. This means there is less space available for the cord and therefore a factor that could predispose to compression and myelopathy.

Genetic conditions such as Down syndrome and Ehlers-Danlos syndrome can predispose to the development of DCM [11]. This is because these patients can have a narrow spinal canal and ligament laxity. Klippel-Feil syndrome is characterised by the congenital fusion of cervical vertebrae which results in accelerated cervical spondylosis and greater biomechanical stress on the adjacent vertebrae [11,16].

Dynamic cord compression can occur during flexion and extension movements of the cervical spine [24]. A cadaveric study by Breig et al. [24] fixed the cervical spines of 42 cadavers in either flexion, extension, or neutral positions. The study used fresh cadaver material which may not accurately reflect the dynamic movement of the spinal cord in a living person. During flexion, the spinal cord is stretched, and its length increases with the opposite occurring during extension. They found that part of the spinal cord became deformed by the mechanical stress produced by spondylotic bars and osteophytes during flexion. This paper supported the theory that DCM is caused by ischaemia of the cord. However, the sample size was small which may have affected the generalisability of the findings. The findings of Breig et al. [24] have been supported in vivo by dynamic MRI studies in patients with DCM [25,26]. These studies are valuable as they explored the effect of dynamic movement on the spinal cord. These changes can put biomechanical stress on the spinal cord and its blood supply, leading to compression and ischaemia. If combined with a stenotic cervical spinal canal the patient is at an increased risk of cord compression due to biomechanical stress [25]. Other dynamic factors include a large cervical range of motion, segmental instability, and spondylolisthesis. It is thought that instability in one part of the cervical spine can cause degenerative changes in adjacent parts [27].

Another component of the pathology of DCM is chronic microtrauma which can occur with day-to-day movement of the spine and is associated with vascular compression and associated ischaemia. There is also a chronic inflammatory response which leads to apoptosis of neurons and oligodendrocytes. This can result in gray matter necrosis, neuronal loss, demyelination, and myelomalacia of the spinal cord [28]. Wallerian degeneration of motor axons in the lateral corticospinal tract can lead to a spastic gait. This is anterograde degeneration of a nerve axon, distal to the site of compression or lesion. Severe DCM presents with proprioceptive defects and sphincter disturbance which is typically caused by degeneration of the central gray matter and the posterior white column [29].

The clinical presentation of degenerative cervical myelopathy

DCM can present with a wide range of signs and symptoms as it depends on the extent and location of compression (Table 2). This often leads to a delay in diagnosis. The most consistent feature of DCM is the progression of symptoms rather than the symptoms present. The most common presenting symptom is paraesthesia in the hands which can be mistaken for carpal tunnel syndrome. Late-stage presentation of DCM includes motor loss, paralysis, and loss of sphincter control [7]. Diagnosis of DCM requires a combination of a clinical history of a myelopathic nature, positive examination findings, and signs of compression on MRI [21]. These clinical findings can be subtle, and MRI findings of compression are not always expressed clinically.

**Table 2:** The signs and symptoms of degenerative cervical myelopathy.

Signs	Symptoms
Lhermitte’s sign (shock) [2,20]	Paraesthesia of the hands [2,7,20,21]
Hoffman’s sign (Flick test) [2,7,20,21]	Loss of finger dexterity [7,21]
Babinski sign [2,7,20,21]	Thenar muscle atrophy [2,20,21]
Romberg sign [2]	Neck pain and stiffness [2]
Inverted brachioradialis reflex [2,21]	Poor coordination [7,21]
Hyperreflexia [2,7,20,21]	Gait disturbance [2,7,20,21]
Lower motor neuron signs (nerve root compression) [21]	Muscle weakness leading to paralysis [2,7,20,21]
	Incontinence, erectile dysfunction [2,7,21]
	Spasticity [2,7,20]
	Clonus [2,7,21]

Imaging

The gold standard imaging modality for diagnosing DCM is MRI which can be used to detect degenerative changes in the cervical spine. This includes changes in soft structures such as the IV discs and spinal ligaments as well as osseous structures such as the vertebral bodies [30,31].

The radiological findings that may be seen include the absence of cerebrospinal fluid (CSF) signal on T2-weighted images, cord signal changes, and T1-weighted imaging hypointensity which indicates cord injury [7]. Cord signal change on MRI can indicate myelomalacia and is typically a result of long-term compression [32,33].

Dynamic MRI is useful to diagnose dynamic cervical cord compression which can often be missed with general imaging. It is a specialised imaging technique that can capture a moving image of the cervical spinal cord and blood vessels during flexion and hyperextension. Dynamic MRI can be more sensitive than static MRI; however, the technique for dynamic MRI is not yet standardised which may lead to confounding factors in studies. The technique also requires specialised equipment and knowledge [25,26].

Cord signal changes on T2-weighted imaging can indicate spinal cord compression, neuronal loss, and myelomalacia [32]. Some studies have identified that the extent of signal change and apparent compression does not always correspond to the clinical presentation of the patient [29,34]. While MRI may indicate severe compression, the patient may not present with expected severe paralysis or sensory disturbance. Thus, MRI can be used to confirm a suspected diagnosis of DCM but should not be considered in isolation.

Myelography is an alternative/adjunct to MRI which involves the injection of a water-soluble dye to highlight a blockage of CSF flow through the spinal canal. Other imaging modalities include flexion/extension radiographs and diffusion tensor imaging (DTI). DTI uses MRI sequences to assess the diffusion of water molecules through tissue producing images of the white matter tracts. This modality is non-invasive and potentially useful to differentiate between DCM severity according to the modified Japanese Orthopaedic Association (mJOA) score [32,35].

Clinical scoring systems for degenerative cervical myelopathy

There are several scoring systems for DCM that surgeons can use to evaluate their patients. The most commonly used scores are the mJOA score (Table 3) and the Nurick score which solely focuses on the lower limb. Another score is the European Myelopathy Score which is similar to the mJOA but also includes proprioception and coordination. The Prolo score focuses on the economic impact on a patient and the last score is the Cooper Myelopathy Scale [36]. The mJOA score ranges from 0 to 18 and classifies a patient with DCM as either mild (15-17), moderate (12-14), or severe (0-11). It considers motor and sensory function in the upper and lower limbs as well as bladder function. The original JOA score was developed in Japan and assessed patients based on their dexterity with chopsticks. It was found to have high inter- and intra-rater reliability; however, this has not been established with the mJOA score [37]. Surgery is generally indicated for those in the moderate and severe categories, and these patients often show significant mJOA score improvement post-surgery. The Nurick score is a scale graded from 0 to 5. It has a low sensitivity as it focuses only on lower limb function despite other key factors for this disease.

**Table 3:** The modified Japanese Orthopaedic Association Score (mJOA) [38].

Category	Score	Description
Motor dysfunction score of the upper extremity /5	0	Inability to move hands
	1	Inability to eat with a spoon but able to move hands
	2	Inability to button a shirt but able to eat with a spoon
	3	Able to button a shirt with great difficulty.
	4	Able to button a shirt with slight difficulty
	5	No dysfunction
Motor dysfunction score of the lower extremity /7	0	Complete loss of motor and sensory function
	1	Sensory preservation without the ability to move the legs
	2	Able to move legs, but unable to walk
	3	Able to walk on flat floor with a walking aid
	4	Able to walk up and/or downstairs with a handrail
	5	Moderate to significant lack of stability but able to walk up and/or downstairs without a handrail
	6	Mild lack of stability but walks with smooth reciprocation unaided
	7	No dysfunction
Sensory dysfunction score of the upper extremities /3	0	Complete loss of hand sensation
	1	Severe sensory loss or pain
	2	Mild sensory loss
	3	No sensory loss
Sphincter dysfunction score /3	0	Inability to micturate voluntarily
	1	Marked difficulty with micturition
	2	Mild to moderate difficulty with micturition
	3	Normal micturition

The management of degenerative cervical myelopathy

Surgery is the preferred treatment for DCM as it can halt disease progression and possibly allow for neurological improvement. Treatment within six months of symptom onset offers the best chance of recovery [39]. However, diagnosis is often longer than this timeframe due to the irregular presentation of DCM [40]. The surgical approach can be anterior, posterior, or combined. An anterior approach can be a corpectomy or anterior cervical discectomy and fusion (ACDF). An anterior approach is generally favoured by surgeons as there are fewer complications [41]. A posterior approach could be a laminectomy, laminectomy with fusion, or laminoplasty [42].

A prospective study by Nagoshi et al. [18] found that Caucasians and East Asians achieved similar gains in their functional status and quality of life scores at 24 months postoperatively. Functional status was assessed using the mJOA and Nurick scores. Quality of life scores included the Neck Disability Index and the Short Form-36 version 2. They also found no significant difference in the rates of perioperative complications between groups and the efficacy of decompressive surgery for DCM was the same. The study controlled for confounding variables; however, a standardised surgical protocol was not used, and there was a 19% attrition rate at 24 months follow-up. Nagoshi et al. [18] accounted for this by using a last observation carried forward approach. A further limitation was the exclusion of other racial cohorts; however, this was due to small patient numbers. Another study compared the functional outcome one year after surgery between Caucasian and African American patients and found no substantial difference in patient-reported outcomes [43]. There are very few studies concerning racial disparities following cervical spinal surgery for DCM. Patients who are younger, have a shorter duration of symptoms, no comorbidities, and a high baseline mJOA score generally do better post-surgery [44]. A systematic review found low-level evidence that patients with DCM who smoke or have coexisting diabetes are at risk of poor postoperative recovery and complications [45]. However, the evidence for the association between smoking, diabetes, and patient outcomes is rather limited, and this area of study requires further research.

Non-operative management includes physical and occupational therapy, neck immobilisation, avoidance of risky activities, and cervical traction [42]. A prospective cohort study of 122 patients conducted across seven centres by Bond et al. [46] reported that surgery is not proven to be superior to conservative treatment for patients in the mild category. This study identified that patients within the mild DCM category with worse quality-of-life scores on EuroQol-5 Dimension questionnaires were more likely to undergo surgical intervention. This study was limited by a small non-operative sample size of 17 patients and may also have suffered from surgeon bias when making treatment decisions.

A systematic review by Rhee et al. [47] also reported that there are similar outcomes in patients with an mJOA score of ≥13 who are treated non-operatively or surgically. However, patients who were managed non-operatively had a rate of hospitalisation for spinal cord injury of 13.9 per 1,000 person-years compared to 9.4 per 1,000 person-years for the surgical group. This would suggest that non-operative treatment may be a suitable option for patients as long as their condition does not progress and deteriorate. With surgical intervention, the risk of progression is much lower as the cervical spine is decompressed and stabilised. Bond et al. [46] and Rhee et al. [47] both report that surgical intervention is not superior to non-operative treatment. However, the study by Bond et al. [46] had a small sample size of patients treated non-operatively, and the study by Rhee et al. [47] may have suffered from selection bias due to the retrospective design. A clinical practice guideline recommends offering patients with mild DCM surgical intervention or a trial of non-operative management; however, this recommendation is weak with low-quality evidence [48]. In clinical practice, surgeons could employ either non-operative management or surgery for patients with mild DCM. A surgeon should consider the individual risk-benefit profile for each patient. There is still a gap in the knowledge of the course of DCM in patients who are treated conservatively and currently no way to identify which patients will deteriorate [32].

Some patients are prescribed Riluzole, a sodium glutamate antagonist that prevents decompression-induced ischaemia-reperfusion injury following surgery [41]. Riluzole appears to reduce the oxidative DNA damage in the spinal cord and preserve corticospinal tract function. However, a phase three trial by Fehlings et al. [49] found no difference in functional recovery between the Riluzole and placebo groups.

Tetreault et al. [50] have developed a clinical prediction rule (CPR) to identify high-risk patients, assess the severity of their disease, and provide prognostic information to patients and clinicians. This CPR provides a percentage likelihood a patient will achieve an mJOA score of >16 after surgery. It identified age, preoperative mJOA score, presence of impaired gait, smoking status, duration of symptoms, and the presence of mental disorders as key clinical predictors of outcomes in cervical spondylotic myelopathy patients. The strength of this study was that the model demonstrated excellent calibration, indicating that the prediction closely matched the actual patient outcome. Managing a patient’s expectation of outcome is particularly important and CPR can be used to give the surgeon and patient an idea of what realistic improvement they could achieve [51]. A limitation of CPR is that the mJOA score has not been validated; however, the JOA score has been validated.

Complications

Complications that can arise after surgery for DCM include dysphagia and hoarseness due to injury of the recurrent laryngeal nerve. Posterior approaches report complications such as instability and kyphosis. Surgeons usually prefer an anterior approach due to the lower incidence of complications [2,52]. A systematic review of 60 studies by Tetreault et al. [53] found that age is the only significant predictor of complications. There was low evidence that patients with ossification of the PLL are at an increased risk of developing a C5 palsy. Furthermore, they found moderate evidence that a longer duration of operation was predictive of overall perioperative complications. Higher rates of neck pain were associated with laminoplasty when compared to anterior fusion, and ACDF was associated with higher rates of dysphagia. The authors also found that diabetes was a significant risk factor for post-surgery wound infections and non-union at the surgical site. A limitation of the study by Tetreault et al. [53] was that only studies published in the English language were included which may exclude relevant papers.

Limitations

A limitation of this overview article is that it provides a general overview of the topic of degenerative cervical myelopathy and the subtopics within it. It may not cover the full depth of literature that concerns DCM.

Recommendations

Future recommendations include research into the prevalence rate of DCM and if there is a difference between populations. Further research on the benefit of conservative management for patients with mild or non-progressive DCM is recommended.

## Conclusions

DCM is an important condition with many researchers devoting time to further our understanding of this disease. Important anatomical structures affected include the vertebral body, IV disc, PLL, LF, and the uncovertebral joints. DCM could present with motor, sensory, and/or sphincter dysfunction and important signs such as Hoffman’s sign and the inverted brachioradialis reflex. Diagnosis is made through clinical examination and supported by evidence of cord compression on MRI. The key functional scoring system is the mJOA score which can be used to assess neurological function before and after surgery. Management of DCM is primarily through surgery, with surgeons favouring the ACDF approach due to the lower rate of complications and less time spent in the hospital after surgery.
